# Contribution to Bone Formation of the Schneiderian Membrane after Sinus Augmentation: A Histological Study in Rabbits

**DOI:** 10.3390/ma15228077

**Published:** 2022-11-15

**Authors:** Su Tien Lim, Kaoru Kusano, Tomohide Taniyama, Shigeru Sakuma, Yasushi Nakajima, Samuel Porfirio Xavier, Shunsuke Baba

**Affiliations:** 1 Department of Oral Implantology, Osaka Dental University, Osaka 573-1121, Japan; 2 Clinical Implant Society of Japan, Tokyo 170-0003, Japan; 3 ARDEC Academy, Viale Giovanni Pascoli 67, 47923 Rimini, Italy; 4 Department of Oral and Maxillofacial Surgery and Periodontology, Faculty of Dentistry of Ribeirão Preto, University of São Paulo, Ribeirão Preto 14040-904, SP, Brazil

**Keywords:** animal study, sinus mucosa, sinus floor elevation, collagen membrane, trap-door, histology, mesenchymal stem cells, periosteum

## Abstract

Aim: to evaluate bone formation in close contact with the sinus mucosa after different periods from sinus augmentation and the influence on healing of the presence of an inward dis-placed bone window. Material and Methods: Eighteen rabbits were included in the experiment. A trap-door technique was applied at the test sites, and the bony window was elevated inward (inward window; IW) together with the sinus mucosa. At the control sites, the bony window was removed before the elevation of the sinus mucosa. The elevated space was filled with deproteinized bovine bone mineral (DBBM) and both access windows were covered with a collagen membrane. Histometric measurements were performed subjacent the sinus mucosa after 2, 4, and 8 weeks of healing. Results: Very few sinuses presented small percentages of new bone in close contact with the sinus mucosa in the various period examined. The presence of bone in the neighbor areas might have influenced bone formation close to the sinus mucosa. The inward displaced bone window supported bone formation close to the sinus mucosa only in the earliest period of healing, while the bone walls increased their influence over time. The lack of increased new bone percentage over time in the most central regions of the elevated sinus mucosa do not support the hypothesis that the sinus mucosa may express its potential in bone formation. It can be speculated that the new bone found in the intermediate and middle regions of the control sites in the earliest period of healing might be due to residual of bone from the osteotomy. Conclusions: Very small amounts of new bone were found subjacent to the sinus mucosa, mostly formed from the bone walls, the inward displaced bone window or from possible bone residues from the osteotomy procedures. The lack of increased new bone percentage over time in the most central regions of the elevated sinus mucosa indicates that the contribution to bone formation provided by the sinus mucosa is limited.

## 1. Introduction

When a sufficient height of alveolar bone crest is not available for implant installation in the posterior segments of the maxilla, sinus floor elevation might be applied to increase bone volume [1].

Bone apposition has been shown to occur over time from the sinus bone walls towards the central regions of the elevated space [2,3,4,5,6,7], while the contribution of the sinus mucosa to bone formation is still under debate. It was shown that the periosteum of the human maxillary tuberosity and jaw contains osteoprogenitor cells capable of inducing ectopic bone formation [8]. Moreover, in an in vitro study [9], sinus mucosa-derived cells were incubated with bone morphogenetic protein (BMP-6 and BMP-7) and, subsequently, the alkaline phosphatase activity, the osteocalcin expression, and the mineralization of the matrix were evaluated. The outcomes allowed to conclude that the sinus mucosa contains progenitor mesenchymal cells that increase their osteogenic differentiation after an exposure to BMP-6 and BMP-7. In another study [10], cultured human maxillary sinus mucosa cells revealed alkaline phosphatase reaction and mRNA expression of osteogenic markers. Fresh tissue samples showed a positive alkaline phosphatase enzyme activity close to the periosteum-like layer. Fresh tissue samples were also transplanted subcutaneously in immunodeficient mice. After 8 weeks, new bone was observed, demonstrating the innate capacity of the sinus mucosa periosteum-like layer of inducing bone formation.

Even though bone formation from the sinus mucosa cells has been clearly demonstrated, the expression of this potential might be limited in clinical situations. In fact, in an experiment in monkeys, it was not possible to show bone formation subjacent the sinus mucosa during the first month of healing [3,11]. In other animal experiments, a collagen membrane was placed subjacent the sinus mucosa only at the test sinus in order to limit a possible bone formation from that site. Conversely, at the control sites, no membranes were placed [6,12,13,14]. The results failed to show differences between test and control groups despite the presence of a collagen membrane at the test sites.

Several experimental studies have reported comparative data of different regions of the elevated space after various periods from sinus augmentation procedure [15,16,17,18,19,20,21]. These studies have reported higher rate of bone formation in regions close to the bone walls compared to the regions subjacent the sinus mucosa. However, those studies evaluated a large region subjacent the sinus mucosa that might have been influenced by other regions. Instead, the aim of the present study was to evaluate bone formation in close contact with the sinus mucosa after different periods from sinus augmentation and the influence on healing of the presence of an inward displaced bone window.

## 2. Materials and Methods

### 2.1. Ethical Statement

The present experiment was performed at the School of Dentistry, Ribeirão Preto, USP, Brazil after the approval obtained from the local Ethical committee (protocol # 2017.1.278.58.9 on 14 June 2017). The ARRIVE checklist, the SYRCLE’s risk of bias tool for animal studies and the Brazilian guidelines as well for animal experiments were followed.

### 2.2. Experimental Animals

Eighteen New Zealand male rabbits of about 4–5 months of age and about 3.4 kg of weight were chosen for the experiment at the Fazenda de coelhos Chacara Uniao, Cidade Taquaritinga, SP, Brasil.

### 2.3. Study Design

The experimental design adopted in the present experiment was a split mouth. Both sinuses were used in the same animal. A trap-door technique was used at the test sites, randomly selected, while at the control sites the bone window was removed. The rabbits were euthanized after 2, 4, and 8 weeks of healing, six animals for each period.

### 2.4. Sample Size

For the present experiment, the data from a previous study were used [5]. In that study, maxillary sinus augmentation was performed bilaterally in rabbit using either DBBM granules or a collagen sponge. At the DBBM site, statistically significant higher bone formation was found in the bone walls regions compared to the sub-mucosa region, especially in the earlier periods of healing (*n* = 5). The data from the intermediate period of 20-day of healing periods were used for calculation, with a mean difference of 15.1 ± 8.8% in new bone between the bone wall and the submucosa regions. A sample composed of six animals each group was calculated to be sufficient to be able to reject the null hypothesis that the response difference was zero with probability 0.90. The Type I error probability was 0.05. The software G*Power 3.1.9.4 was used for calculation.

### 2.5. Randomization and Allocation Concealment

A digital randomization of the treatments was performed by one professional that did not participate to the surgery. Sealed and opaque envelopes containing the allocation treatment were prepared and were opened after the preparation of the access window by a professional not involved in the surgery.

### 2.6. Experimental Procedures

For more details see a previous published paper on the same experiment that reported the data on the healing inside the sinus, but not analyzing the tissues in close contact with the sinus mucosa (Amari et al.) [17]. Briefly, the anesthesia of the rabbits was performed with acepromazine (1.0 mg/kg; Acepran^®^, Vetnil, Louveira, São Paulo, Brazil) injected subcutaneously, and xylazine (3.0 mg/Kg; Dopaser^®^, Hertape Calier, Juatuba, Minas Gerais, Brazil) and ketamine hydrochloride (50 mg/kg; Ketamin Agener, União Química Farmacêutica Nacional S/A, Embu-Guaçú, São Paulo, Brazil) injected intramuscularly. Local anesthesia was also provided.

The surgical procedures were carried out by an expert maxillofacial surgeon (see the acknowledgments and the previous publication). The skin of the nasal dorsum was incised, and the flaps were elevated to expose the subjacent nasal bone. Access window, 3 mm wide and 4 mm in height, were prepared about 1 cm anteriorly to the frontal-nasal suture, and laterally to the nasal-incisal suture. In the test group the window was displaced inward during the elevation of the sinus mucosa while at the control sites the bone window was removed. Similar volumes of deproteinized bovine bone mineral (DBBM; Bio-Oss^®^, granules 0.250–1.0 mm, Geistlich Biomaterials, Wolhusen, LU, Switzerland) were grafted within the elevated space. A collagen membrane (Bio-Gide^®^, Geistlich Biomaterials, Wolhusen, LU, Switzerland) was used to cover both access window.

### 2.7. Housing and Husbandry

The animals were maintained in individual cages in rooms with controlled light and temperature at the experimental facilities of the School of Dentistry, USP, Ribeirão Preto (Brazil). The animals were monitored for pain, infection and biological functions during the full period of the experiment. A prophylactic dose of oxytetracycline dehydrate (40 mg/kg, intramuscular was injected to all animals, Terramicina LA, Zoetis Indústria e Produtos Veterinários, Campinas, Sao Paulo, Brazil). Postoperatively, ketoprofen (3.0 mg/kg, IM, Ketofen, Merial, Monte-Mor, Sao Paulo, Brazil) and tramadol hydrochloride subcutaneously (Tramadol 2%, 1.0 mg/kg, Cronidor, Agener União Saúde Animal, Apucarana, Parana, Brazil) were administrated.

### 2.8. Euthanasia

The animals were anesthetized and were subsequently euthanized by a lethal dose of sodium thiopental (1.0 g, 2 mL, Thiopentax^®^, Cristália Produtos Químicos Farmacêuticos, Itapira, São Paulo, Brazil). Biopsies were obtained in blocks and fixed in 10% buffered formalin.

### 2.9. Histological Preparation

The biopsies were dehydrated and embedded in resin (LR white hard grid, London Resin, Agar Scientific Ltd., Stansted, Essex, UK) and, after the polymerization, two ground sections representing the most central zone of the sinuses were prepared using a cutting-grinding equipment (Exakt; Apparatebau, Norderstedt, Germany). The slides were stained with either Stevenel’s blue and alizarin red or toluidine blue.

### 2.10. Histological Analyses

**Figure 1:**
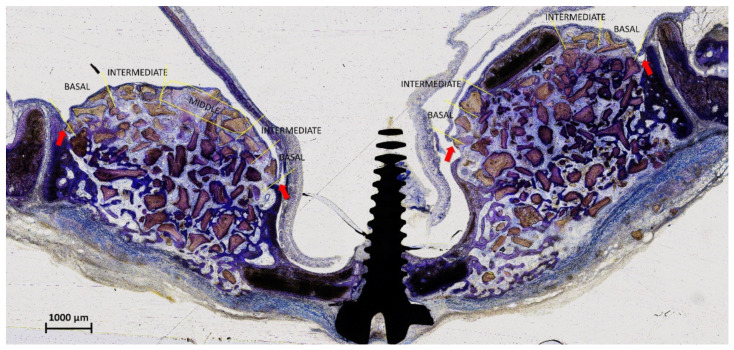
The sinus mucosa included between the sinus walls and the window displaced inward (inward window; IW) was divided into two parts, both at the lateral and medial sides: one part towards the bone walls (basal region) and the other part towards the IW (intermediate region). At the control site, the contour of the IW of the text site was drawn and superposed on a similar position at the control sites and similar regions to those selected at the test sites were identified. Moreover, in the control sites, also the region delimited by the superposed window contour was identified (middle region). Th red arrows indicate the mucosal folding region (MFR).

The elevated sinus mucosa was divided into 5 regions (Figure 1). The Middle region included the window displaced inward (inward window; IW) at the test sites. The contour of the IW was drawn and superposed on a similar position at the control sites to identify the Middle region in that sinus. The mucosa included between the middle region and the mucosal folding region (MFR) was divided into two regions at both the medial and lateral sides, i.e., the basal, close to the MFR, and the intermediate, located between the basal and the middle regions.

Measurements at ×200 magnification were made on a microscope connected with a camera to a computer. The whole length of the mucosa was evaluated. The software NIS-Elements D 5.11 (Laboratory Imaging, Nikon Corporation, Tokyo, Japan) was used for histological evaluation.

The linear measurements were performed at the base of the sinus mucosa in all five regions at both test and control sites. The following tissues in close contact with the sinus mucosa were measured with a linear method in each identified region: new bone, xenograft, and soft tissues. The percentage of each tissue was obtained based on the total length of the mucosa assessed. Mean values were obtained between regions at the medial and lateral sides of the sinus. At the middle region of the test sites, new bone was evaluated.

The morphometric measurements were performed in a region within 200 µm from the sinus mucosa. A point counting procedure was adopted and carried out using a lattice with squares of 50 microns in dimensions superposed to the images. All regions were evaluated apart from that middle of the test sites.

### 2.11. Calibration for Histomorphometric Evaluations

An expert examiner performed all histological assessments (D.B.; see acknowledgments), after having performed a calibration that resulted in a K > 0.90 intra-rater agreement. Two evaluations, performed in different periods, of several images randomly taken from different histological slides were performed. Differences in percentages for both linear and morphometric assessments were assessed. Similar methods and magnifications used for the histological measurements were adopted.

### 2.12. Experimental Outcomes

Both histological slides were analyzed, and the mean values calculated. The primary variable for both linear and morphometric measurements was new bone percentage in the various regions analyzed. As secondary variable, the xenograft percentage was used.

### 2.13. Statistical Methods

The Shapiro-Wilk test was applied to assess the normal distribution and either a paired *t* test or a Wilcoxon test was used to evaluate differences between test and control in the various regions evaluated. Furthermore, for exploratory purposes, the percentages of new bone in the various regions evaluated were compared with those in the region near the bone walls, reported in a previous evaluation of the same material [17]. Difference between periods were evaluated applying the Kruskal-Wallis test followed by an unpaired *t* test or a Mann-Whitney test. The software GraphPad Prism (version 9.4.1 for Windows, GraphPad Software, San Diego, CA, USA) was used for statistical analyses. An α = 5% was applied.

## 3. Results

### 3.1. Tissues in Contact with the Sinus Mucosa; Linear Measurements

**Figure 2:**
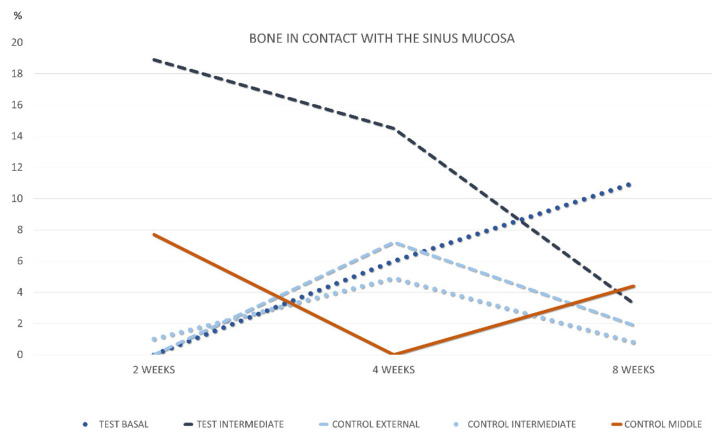
Graph representing the percentage of new bone in contact with the sinus mucosa in the various regions evaluated after 2, 4, and 8 weeks of healing.

After 2 weeks of healing, no new bone was found in contact with the sinus mucosa in the basal regions in both groups (Table 1; Figure 2).

**Figure 3:**
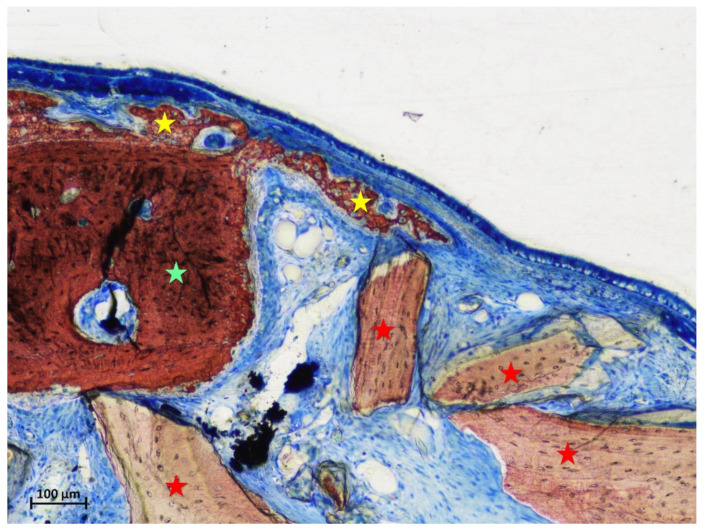
Photomicrograph of a ground section at the intermediate region of a test site after 2 weeks of healing. New bone was found always connected to the bone window. Stars indicate example of new bone (yellow) and xenograft granules (red). Stevenel’s blue and alizarin red stain.

In the intermediate region of the test group, a fraction of 18.9% of new bone was found, in all cases in continuity with the bone window (Figure 3).

**Figure 4:**
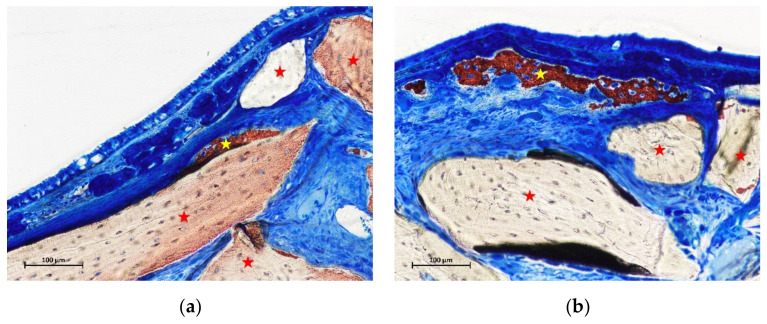
(**a**) photomicrograph of a ground section at the intermediate region of a control site after 2 weeks of healing. Small amounts of new bone lining part of the xenograft surface. (**b**) photomicrograph of a ground section at the middle region of a control site after 2 weeks of healing. New bone was lining a region subjacent the sinus mucosa without any contact with the neighbor xenograft. Stars indicate example of new bone (yellow) and xenograft granules (red). Stevenel’s blue and alizarin red stain.

In the control sites, small amounts of new bone were found in one sinus in the intermediate region (Figure 4a), and in two sinuses in the middle regions (Figure 4b). New bone was found formed on the inward bone window (IW), between the old bone and the sinus mucosa, covering a proportion of 67.8% of the length of IW. The pseudo-periosteum was clearly visible on the top of the inward bone window and on the newly formed bone (Figure 3 and Figure 4b) while its structure was lost in most of the other regions of the elevated mucosa in both test and control sites. However, a dense tissue was often observed between xenograft particles and sinus mucosa (Figure 4a).

**Figure 5:**
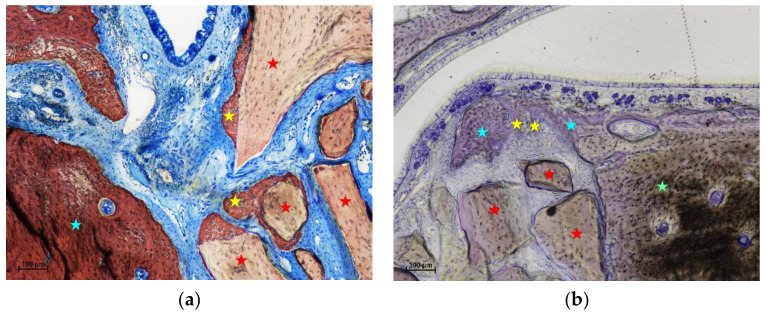
(**a**) photomicrograph of a ground section at the base region of a test site after 4 weeks of healing. The new bone was mainly in connection with the sinus bone walls. (**b**) photomicrograph of a ground section at the base region of a test site after 4 weeks of healing. Note new bone (yellow stars) formed from remnants of old bone (light blue stars) fractured together the bone window (light green star). Some xenograft granules are indicated in red. a, Stevenel’s blue and alizarin red stain; b, Toluidine blue stain.

After 4 weeks of healing, new bone was found in the basal regions in both test and control sinuses, at proportion of 6.0% and 7.2%, respectively (Table 2 and Figure 2). The new bone was mainly in connection with the sinus bone walls (Figure 5a). In the intermediate region of the test sites, new bone was 14.5%, in all cases as extension of the bone window (Figure 5b). At the control sites, only one sinus presented some new bone in the intermediate region, while no new bone was found in the middle region in any control sinus. On the inward bone window, new bone was found in proportion of 33.8%. The pseudo-periosteum presented similar characteristic as in the 2-week healing period.

After 8 weeks of healing, in the basal region, new bone was present in lower percentages and in continuity with the bone walls in both test and control sites, being 7.1% and 1.9%, respectively (Table 3 and Figure 2). Little bone was present in the intermediate region of the test sites, again in connection of the bone window. New bone was found in the intermediate zone in two sinuses, and in one sinus in the middle region of the control sites. On the inward bone window, new bone decreased to the proportion of 19.1%, being the difference with the 2-week period statistically significant. The pseudo-periosteum shown similar features as observed in the previous periods of healing.

**Figure 6:**
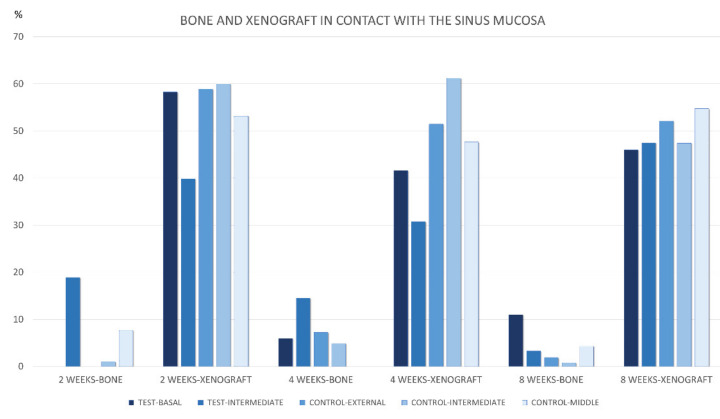
Graph representing the percentage of new bone and xenograft in contact with the sinus mucosa in the various regions evaluated after 2, 4, and 8 weeks of healing.

The percentage of the xenograft in contact with the sinus mucosa was higher compared to that of new bone at the corresponding regions in all periods examined (Figure 6). Only in the intermediate region the difference did not reach a statical significance. In the 8-week period, the new bone formed on the graft particles was mainly located in the opposite side in relation to the sinus mucosa.

### 3.2. Morphometric Measurements

**Figure 7:**
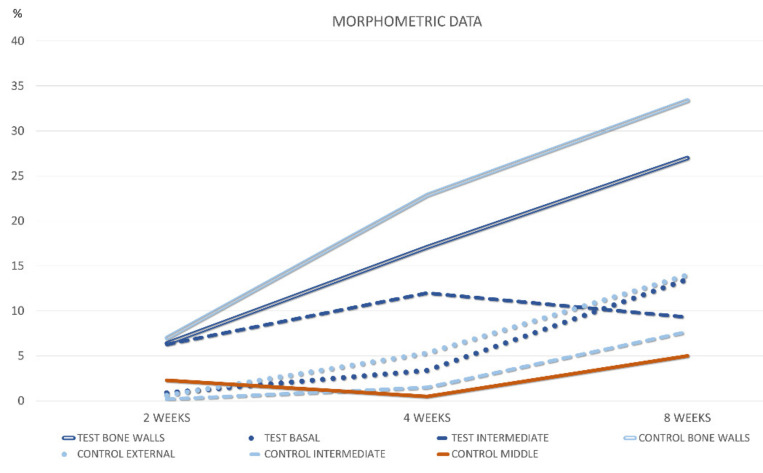
Graph representing new bone density in the various regions subjacent the sinus mucosa after 2, 4, and 8 weeks of healing.

After 2 weeks (Table 4; Figure 7), proportion >1% of new bone was only found in the intermediate region of all test sites (mean 6.3%) and in the middle region of three control sites (mean 2.3%).

**Figure 8:**
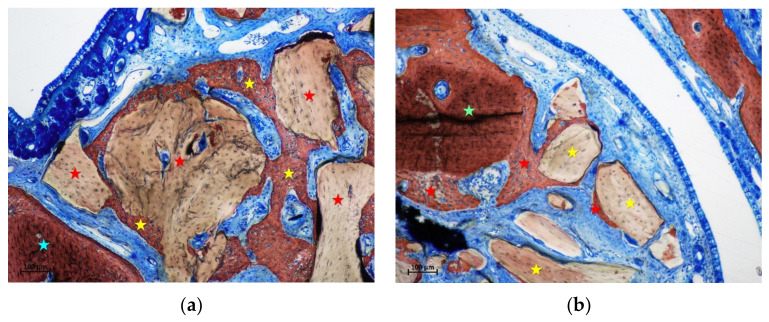
(**a**) photomicrograph of a ground section at the basal region of a control site after 4 weeks of healing. New bone was lining the DBBM surface and in close continuity with the sinus bone walls. (**b**) photomicrograph of a ground section at the intermediate region of a test site after 4 weeks of healing. New bone increased at the test sites, mainly for the contribution of the bone window. Stars indicate example of new bone (yellow), old bone (light blue), inward bone window (light green), and xenogeneic granules (red). Stevenel’s blue and alizarin red stain.

**Figure 9:**
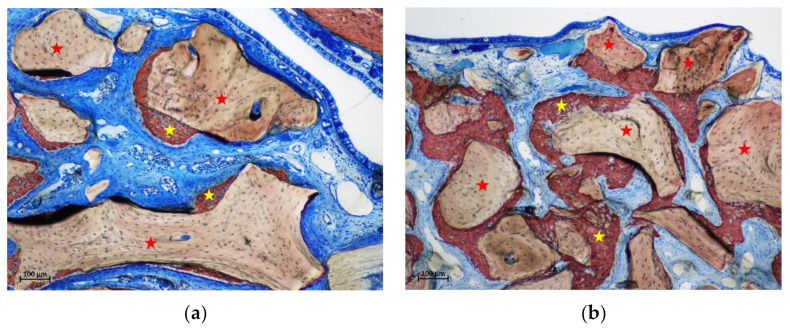
(**a**) photomicrograph of a ground section at the intermediate region of a test site after 4 weeks of healing. Note new bone formed on the xenograft surfaces opposite the sinus mucosa. (**b**) photomicrograph of a ground section at the middle region of a control site after 8 weeks of healing. New bone presented a continuity with that formed in the other regions of the elevated space. Stars indicate example of new bone (yellow), and xenogeneic granules (red). Stevenel’s blue and alizarin red stain.

After 4 weeks of healing (Table 5; Figure 7), new bone increased in all sites, with the exclusion of the middle region of the control site, that presented only one sinus with new bone (mean 0.5%). New bone appeared to be in connection with the bone walls and the inward bone window (Figure 8a,b). The difference between the two intermediate regions was statistically significant. When the comparisons were made with the bone wall fractions of new bone, the differences were statistically significant with all regions in both groups, excluding the intermediate at the test sites. In several sites, new bone was observed forming onto the opposite surface of the biomaterial in relation to the sinus mucosa (Figure 9a).

After 8 weeks, new bone increased in percentages, presenting however higher values at the basal compared to the middle regions. (Table 6 and Figure 7). The differences with the bone walls in new bone formation were all statistically significant. In the intermediate and middle regions of the control sites, new bone presented a continuity with that formed in the other regions of the elevated space (Figure 9b). Moreover, new bone presented the tendency to be formed on the opposite surface of the xenograft in respect to the sinus mucosa (Figure 9b). The xenograft proportion difference between 2 and 8 weeks of healing was statistically significant only in the bone walls regions, while in the other regions minor changes were registered.

## 4. Discussion

The aim of the present study was to analyze bone formation in close contact with the sinus mucosa after different periods from sinus augmentation.

Several previous studies have evaluated differences between the various regions within the elevated space [15,16,17,18,19,20,21]. However, the tissues in close contact with the sinus mucosa were not properly analyzed yet.

For the analysis, a similar method used for the assessment of bone-to-implant contact was adopted. This means that tissues and graft in contact with the mucosa were assesses and the fraction in relation to the length of the region of the elevated sinus mucosa evaluated was subsequently calculated. Moreover, the tissues content in an area subjacent the sinus mucosa was also evaluated to include an area up to which the influence of the sinus mucosa potential might express its potential to form bone.

Different regions of the sinus mucosa were assessed to allow an evaluation of different factors that might have influenced bone formation. The present design was selected because the presence of the trap door displaced inward the subantral space together the mucosa has been shown to influence bone formation in this area [17]. Similar considerations might be done for the regions of the elevated sinus mucosa close to the bone walls.

After 2 weeks of healing, the proximity to the sinus bone walls did not influence bone formation in that region in both groups. However, the sinus mucosa as well did not show any production itself of new bone in the region close to the sinus bone walls. In the intermediate regions of the sinus mucosa, that were those located in proximity of the displaced bone window, presented a quite high rate of new bone formation. New bone was in connection with the bone window so that its influence on bone production might be supposed. On the contrary, in the same intermediate region at the contralateral control sinus, very little new bone was observed (1.0%). In the middle region of the control sites, higher amounts of new bone were found in a couple of sinuses. In one sinus, new bone was subjacent the submucosa with no contact with the xenograft granules (Figure 5). In that region, no other new bone was found in the neighbor areas so that it might be supposed that bone formation was not influenced by that of other regions. In the second sinus, new bone was found formed on the xenograft surface. This provides support to the hypothesis that new bone can be formed from the sinus mucosa. However, it might be speculated that a thin residual layer of bone could have been left attached to the sinus mucosa during surgery, especially close to the edges of osteotomy where a diamond drill was used for preparation of the access window (Figure 7). This residual bone might have triggered the formation on new bone. As support to this speculation might be the lack of new bone formation in the basal regions of the sinus mucosa, that are those regions not involved by the drill during the antrostomy preparation and just detached from the lateral and medial sinus walls with curettes. Moreover, only one sinus in the intermediate region, and two sinuses in the middle regions of the control sites presented few amounts of bone while all the others were devoid of newly formed bone.

Assuming that the sinus mucosa could induce bone formation, after 4 and 8 weeks of healing there should have been an increase in the percentage of new bone in contact with the sinus mucosa. Instead, after 8 weeks of healing, only the basal regions close to the bone walls showed a slight increase in percentages, while the other regions presented a decreased fraction of new bone compared to the 2-week period. No statistically significant differences in bone percentages were found between 2 and 8 weeks of healing in any of the regions evaluated, with the exclusion of the intermediate and middle regions at the text site that lost bone significantly. The decreased percentage of new bone over time observed in the interface between the inward bone window and the sinus mucosa might be interpreted as due to the pressure inside the sinus. This pressure might have triggered bone resorption, as already shown in previous experiments in sinus floor elevation in monkeys and rabbits [3,22,23]. Moreover, the integration of new bone to the biomaterial grafts was mainly located on the opposite surfaces in relation to the sinus mucosa. It has also to be considered that a clear structure of the pseudo-periosteum in the elevated mucosa could only be observed on the top of the inward bone window. The remaining elevated sinus mucosa did not show the presence of pseudo-periosteum. The lack of presence of the pseudo-periosteum might explain the lack of a sound bone formation from the sinus mucosa, even though the presence of mesenchymal cells with the potential of forming bone cannot be excluded.

In the morphometric analyses, after 8 weeks of healing, the differences between new bone formed at the sinus walls regions was higher compared to all the regions evaluated at the sinus mucosa. The increased percentage in the basal and intermediate regions between 2 and 8 weeks appeared to be due to the contribution of the sinus walls to bone formation.

The xenograft used in the present study possesses a high osteoconductivity and new bone was found formed onto the graft surfaces in all regions of the elevated area [5,24]. However, despite this property, only small proportions of xenograft in contact with the sinus mucosa were covered by bone while a high percentage of xenograft was in direct contact with the mucosa without any new bone interposed. This outcome does not support the hypothesis that the mucosa might participate to bone formation. The elevated space presents a tendency to lose volume over time so that new bone already formed during the first periods of healing showed signs of resorption afterwards [3,22,23]. Moreover, after sinus floor elevation, post-surgical bleeding and edema will invade the space included between the sinus mucosa and the xenograft [3,25,26,27,28] so that it seems unlikely that the sinus mucosa might participate to bone formation, at least during this first period of healing. The sinus mucosa also suffers of several thinning phenomena and perforations during healing when similar xenografts were used as fillers [29,30,31], circumstance that might affect its bone formation potential. It should be moreover considered that experimental studies failed to show differences in bone formation between sinuses with a collagen membrane placed subjacent the sinus mucosa and control sinuses with no membranes [6,12,13,14].

It might be summarized that that the presence of new bone subjacent the sinus mucosa in the early stages of healing might suggest that the sinus mucosa can participate to bone formation. However, very few sinuses presented small percentages of new bone in close contact with the sinus mucosa. The presence of bone in the neighbor areas might influence bone formation close to the sinus mucosa. The inward displaced bone window provided an influence in bone formation close to the sinus mucosa only in the earliest period of healing, while the bone walls increased their influence over time. The lack of increased new bone percentage over time in the most central regions of the elevated sinus mucosa, as assessed both in the histometric and morphometrical analyses, do not support the hypothesis that the sinus mucosa may express its potential in bone formation. It can be speculated that the new bone found in the intermediate and middle regions of the control sites might be due to residual of bone from the osteotomy preparation and detachment of the bone window.

As limitations of the present study should be included the low sample used for each period of healing, the smaller dimensions of the sinuses, and thinner sinus mucosa width of the animal model used compared to humans [6,32,33]. The present experiment only evaluated the initial period of healing in which the contribution of the sinus mucosa in bone formation was not substantiated by the outcomes. Longer periods of healing should be allowed to verify a possible contribution of the sinus mucosa on the corticalization of the new sinus floor subjacent the elevated sinus mucosa. Moreover, a study that compares a xenograft vs. autogenous bone might be carry out to disclose differences between them in influencing bone formation from the sinus mucosa. However, the sinus mucosa might be thin in several individuals [32,33]. The faster rate of healing in animals compared to humans should be also considered [34]. Hence, any inference with humans should consider these limitations.

The most important findings of the present study resulted from the evaluation of bone formation in close contact with the sinus mucosa. This allowed to observe that new bone in that region, instead of increasing in percentage over time, was found decreased between 2 and 8 weeks. New bone was found formed in high percentage between the inward bone window and the sinus mucosa after 2 weeks. However, the percentage decreased over time also in this region. A sound pseudo-periosteum was only identified at the top of the inward bone window while its structure was lost in most of the other regions of the elevated mucosa.

More studies should be performed with a larger sample, specifically directed to study bone formation from the sinus mucosa, and the presence of cells with the potential of forming bone in the elevated sinus mucosa.

## 5. Conclusions

In conclusion, very small amounts of new bone were found subjacent to the sinus mucosa, mostly formed from the bone walls, the inward displaced bone window or from possible bone residues from the osteotomy procedures. The lack of increased new bone percentage over time in the most central regions of the elevated sinus mucosa indicates that the contribution to bone formation provided by the sinus mucosa is limited.

## Figures and Tables

**Table 1:** Linear measurements. Tissues in contact with the sinus mucosa after 2 weeks of healing in the various regions evaluated. Mean values ± standard deviation in percentages. Test sites with the inward window.

	Test Sites			Control Sites		
	Basal	Intermediate	Middle	Basal	Intermediate	Middle
New bone	0.0 ± 0.0 ^b,c^	18.9 ± 15.0 ^a,b,c^	67.8 ± 12.6 ^a^	0.0 ± 0.0 ^b^	1.0 ± 2.6 ^a,b^	7.7 ± 17.4 ^a,b^
Xenograft	58.3 ± 14.4 ^b,c^	39.8 ± 5.8 ^a,b,c^	-	58.9 ± 12.9 ^b^	60.0 ± 12.9 ^a,b^	53.1 ± 19.4 ^b^
Soft tissue	41.7 ± 14.4	41.2 ± 13.2	-	41.1 ± 12.9	39.0 ± 13.2	39.2 ± 15.3

^a^, *p* < 0.05 between test and control; ^b^, *p* < 0.05 between new bone and xenograft, within the same group; ^c^, *p* < 0.05 between basal and intermediate regions, within the same group.

**Table 2:** Linear measurements. Tissues in contact with the sinus mucosa after 4 weeks of healing in the various regions evaluated. Mean values ± standard deviation in percentages. Test sites with the inward window.

	Test Sites			Control Sites		
	Basal	Intermediate	Middle	Basal	Intermediate	Middle
New bone	6.0 ± 9.4 ^b^	14.5 ± 15.0 ^b^	33.8 ± 29.3 ^a^	7.2 ± 11.8 ^b^	4.9 ± 12.0 ^b^	0.0 ± 0.0 ^a,b^
Xenograft	41.6 ± 15.6 ^b^	30.8 ± 11.7 ^a,b^	-	51.4 ± 25.7 ^b^	61.2 ± 16.2 ^a,b^	47.7 ± 9.1 ^b^
Soft tissue	52.4 ± 22.4	54.7 ± 17.9	-	41.3 ± 19.7	33.9 ± 10.5	52.3 ± 9.1

^a^, *p* < 0.05 between test and control; ^b^, *p* < 0.05 between new bone and xenograft, within the same group.

**Table 3:** Linear measurements. Tissues in contact with the sinus mucosa after 8 weeks of healing in the various regions evaluated. Mean values ± standard deviation in percentages. Test sites with the inward window.

	Test Sites			Control Sites		
	Basal	Intermediate	Middle	Basal	Intermediate	Middle
New bone	7.1 ± 13.7 ^b^	3.3 ± 4.2 ^b^	19.1 ± 19.3 ^a^	1.9 ± 3.0 ^b^	0.8 ± 1.5 ^b^	4.4 ± 10.7 ^a,b^
Xenograft	50.2 ± 12.3 ^b^	47.5 ± 14.5 ^b^	-	52.1 ± 16.6 ^b^	47.4 ± 20.5 ^b^	54.7 ± 14.8 ^b^
Soft tissue	42.7 ± 17.1	49.2 ± 14.8	-	46.0 ± 14.4	51.8 ± 21.5	40.9 ± 9.9

^a^, *p* < 0.05 between test and control; ^b^, *p* < 0.05 between new bone and xenograft within the same group. No statistically significant differences between test and control sites.

**Table 4:** Morphometric evaluation of the tissues after 2 weeks of healing in the various regions evaluated. Mean values ± standard deviation in percentages. Test sites with the inward window.

	Test Sites			Control Sites			
	Bone Walls	Basal	Intermediate	Bone Walls	Basal	Intermediate	Middle
New bone	* 6.4 ± 5.1 ^b^	0.9 ± 2.3 ^b^	6.3 ± 2.3 ^a^	7.0 ± 6.9 ^b,c^	0.6 ± 0.9 ^b^	0.2 ± 0.5 ^a,c^	2.3 ± 2.8
Xenograft	* 47.9 ± 3.7 ^b,c^	45.5 ± 6.1 ^a,b^	39.7 ± 7.8 ^c^	45.9 ± 54.9 ^b^	54.4 ± 7.9 ^a,b^	51.1 ± 10.5	46.9 ± 13.3
Soft tissue	* 43.9 ± 5.7 ^b,c^	62.3 ± 5.6 ^a,b^	60.3 ± 10.8 ^c^	47.1 ± 2.8	52.4 ± 9.0 ^a^	56.7 ± 12.5	59.5 ± 14.5

^a^, *p* < 0.05 between test and control; ^b^, *p* < 0.05 between bone walls and basal region, within the same group; ^c^, *p* < 0.05 between bone walls and intermediate region, within the same group. * Data already reported in a previous published study (Amari).

**Table 5:** Morphometric evaluation of the tissues after 4 weeks of healing in the various regions evaluated. Mean values ± standard deviation in percentages. Test sites with the inward window.

	Test Sites			Control Sites			
	Bone Walls	Basal	Intermediate	Bone Walls	Basal	Intermediate	Middle
New bone	* 17.1 ± 8.9 ^a,b^	3.4 ± 3.8 ^b^	12.0 ± 9.3 ^a^	22.9 ± 8.7 ^a,b,c,d^	5.3 ± 6.1 ^b^	1.5 ± 2.1 ^a,c^	0.5 ±1.2 ^d^
Xenograft	* 42.7 ± 8.6	39.3 ± 8.2	37.8 ± 7.9	41.6 ± 7.2	38.3 ± 7.1	44.9 ± 8.0	46.9 ±5.1
Soft tissue	* 40.2 ± 5.6 ^b,c^	67.0 ± 6.8 ^b^	58.4 ± 10.7 ^c^	35.5 ± 4.5 ^b,c,d^	65.4 ± 4.5 ^b^	62.4 ± 9.6 ^c^	61.5 ±6.3 ^d^

^a^, *p* < 0.05 between test and control; ^b^, *p* < 0.05 between bone walls and basal region, within the same group; ^c^, *p* < 0.05 between bone walls and intermediate region, within the same group; ^d^, *p* < 0.05 between bone walls and middle region in the control group. * Data already reported in a previous published study (Amari).

**Table 6:** Morphometric evaluation of the tissues after 8 weeks of healing in the various regions evaluated. Mean values ± standard deviation in percentages. Test sites with the inward window.

	Test Sites			Control Sites			
	Bone Walls	Basal	Intermediate	Bone Walls	Basal	Intermediate	Middle
New bone	* 27.0 ± 10.3 ^a,b,c^	13.5 ± 7.3 ^b^	9.3 ± 10.5 ^c^	33.4 ± 4.2 ^a,b,c,d^	14.0 ± 4.0 ^b^	7.7 ± 8.5 ^c^	5.0 ± 5.9 ^d^
Xenograft	* 36.8 ± 9.5 ^b^	42.8 ± 8.9 ^b^	41.2 ± 11.1	36.0 ± 4.1 ^d^	45.6 ± 7.0	46.3 ± 10.3	49.0 ± 8.2 ^d^
Soft tissue	* 36.2 ± 10.3 ^b,c^	50.0 ± 9.5 ^b^	54.3 ± 7.5 ^c^	30.6 ± 2.8 ^b,c,d^	48.8 ± 15.0 ^b^	52.9 ± 7.4 ^c^	52.4 ± 11.3 ^d^

^a^, *p* < 0.05 between test and control; ^b^, *p* < 0.05 between bone walls and basal region, within the same group; ^c^, *p* < 0.05 between bone walls and intermediate region, within the same group; ^d^, *p* < 0.05 between bone walls and middle region in the control group. * Data from a previous published study and reported for comparison (Amari).

## Data Availability

Data is available under reasonable request.
